# Can knowledge-based autoplanning keep up with advances in radiotherapy optimization for oropharyngeal cancer?

**DOI:** 10.3389/fonc.2026.1755913

**Published:** 2026-03-17

**Authors:** Vanessa Panettieri, Olubunmi Olumuyiwa, Lars Södergren, Julia Söderström, Mimmi-Carolin Bolin, Marianne Falk, Anna Embring, Eva Onjukka

**Affiliations:** 1 Department of Radiation Oncology, Peter MacCallum Cancer Centre, Melbourne, VIC, Australia; 2 Sir Peter MacCallum Department of Oncology, The University of Melbourne, Melbourne, VIC, Australia; 3 School of Translational Medicine, Monash University, Melbourne, VIC, Australia; 4 Department of Medical Imaging and Radiation Sciences, Monash University, Clayton, VIC, Australia; 5 Department of Physics, Stockholm University, Stockholm, Sweden; 6 Department of Nuclear Medicine and Medical Physics, Karolinska University Hospital, Stockholm, Sweden; 7 Department of Oncology Pathology, Karolinska Institutet, Stockholm, Sweden; 8 Department of Radiotherapy, Karolinska University Hospital, Stockholm, Sweden

**Keywords:** automated planning, head and neck cancer, hybrid IMRT, normal-tissue complication probability, rapidarc dynamic

## Abstract

**Introduction:**

The performance of knowledge-based autoplanning depends on historical plans used to train the autoplanning models. Here, we investigate the applicability of an existing RapidPlan (RP) model for head and neck cancer to a novel planning and delivery solution (RapidArc Dynamic, RAD), which has the potential to improve plan quality through the integration of new degrees of freedom. RAD integrates static-angle modulated ports and a dynamic collimator in volumetric-modulated arc therapy (VMAT) fields.

**Materials and methods:**

A cohort of 48 oropharyngeal cancer (OPC) patients was retrospectively included in the planning study. Organ-at-risk (OAR) sparing was evaluated for VMAT and RAD, respectively, using both a VMAT-based RP model and a RAD-based RP model, resulting in four plans per patient: VMAT (RP-VMAT), VMAT (RP-RAD)), RAD (RP-VMAT) and RAD (RP-RAD). Differences were assessed with the related-samples Friedman’s two-way ANOVA test by ranks, correcting the p value for multiple testing (p ≤ 0.05 considered significant).

**Results:**

For RAD plans, the RAD-based RP model improved the sparing of all OAR (p ≤ 0.001) except the parotids. However, the RAD plans were at least equivalent to the VMAT plans when optimizing with RP-VMAT, indicating the safety of initially implementing RAD with a VMAT-based RP model. In addition, when optimizing with RP-RAD, all OAR except the trachea were significantly better spared with RAD (RP-RAD) compared to VMAT (RP-RAD) (p = 0.039 for the esophagus and < 0.001 for the remaining OAR), with a median reduction of Dmean by 4.8 Gy and 3.5 Gy, respectively, for the larynx and the constrictor muscle. There was also a significant reduction in the estimated risk of dysphagia (1.9 pp) and acute mucositis (1.3 pp) (p ≤ 0.001).

**Conclusions:**

VMAT-based RP models appear to remain applicable for optimization with the novel RAD solution until RAD-specific RP models are developed. Furthermore, RAD shows promise for OPC in terms of sparing of midline OARs.

## 1 Introduction

Radiation therapy (RT), with and without chemotherapy, is a well-established strategy used in the definitive treatment of head and neck cancers (HNC) in general, and oropharyngeal cancer (OPC) specifically (1). This anatomical site usually represents a complex treatment case, due to the proximity of multiple organs-at-risk (OARs) and has benefited from technological advances such as intensity-modulated RT (IMRT) and volumetric modulated arc therapy (VMAT) (2–8). Several HNC planning studies have reported that VMAT can provide adequate normal tissue sparing and tumor coverage while reducing delivery time in comparison to other techniques, making it a popular solution in many centers worldwide (4, 7).

In parallel, autoplanning has been adopted in clinical practice for HNC, to improve efficiency and consistently provide plans equivalent to those obtained by experienced planners, reducing the user-experience dependence of plan quality (9–13). Centers have reported use of autoplanning in more than 40% of their clinical cases overall, making this an essential part of their clinical practice (14).

While the complexity of treatment plans has increased, currently available VMAT optimization algorithms use methods originally developed as far back as in 2008 with the introduction of the RapidArc solution (Varian Medical Systems, Palo Alto, USA). In recent years, it has become evident that these algorithms might not exploit all the available degrees of freedom of the modern delivery machines, due to their limited evolution (15). To address these limitations, several investigations have explored the use of hybrid IMRT/VMAT techniques capable of exploiting the advantages of both modalities (namely the delivery efficiency of VMAT arcs and the localized intensity modulation of IMRT), based on sequential (16) and comprehensive techniques (17). Extending from such experience, the novel RapidArc Dynamic (RAD) solution (Varian Medical Systems, Palo Alto, USA) has been developed, integrating features such as IMRT/VMAT hybrid planning, and a dynamically rotating collimator (18–21). The hybrid feature has been implemented through *static angle modulated ports* (STAMPs), enabling the delivery of some control points at a fixed gantry angle, at selected positions throughout an arc field. This solution offers more degrees of freedom compared to the VMAT optimization algorithm and potentially enhances the ability to sculpt the dose in challenging HNC cases.

While the introduction of RAD might allow more flexibility in the planning process, its adoption presents the challenge of replacing established protocols and planning approaches. The autoplanning solution offered by Varian uses the knowledge-based technique, which requires training on a large cohort before clinical use. As a consequence, centers that rely heavily on the use of knowledge-based autoplanning will have to assess if their current models are applicable to RAD planning, or if re-training based on plans generated with the new solution will be required. The main purpose of this study is to address this question, through comparing the plan quality for OPC RAD plans optimized with 1) a RapidPlan (RP) model (9, 13) trained on VMAT plans and 2) a model trained on RAD plans. In addition, the potential of the added degrees of freedom for OAR sparing will be evaluated by comparing the plan quality for RAD- and VMAT plans, respectively, both optimized with RP.

## 2 Materials and methods

### 2.1 Study cohort

A retrospective planning study was performed in Eclipse version 18.1, comparing plan quality resulting from the use of two RP models. The first model (RP-VMAT) is currently in clinical use for VMAT planning techniques and was trained on plans optimized with VMAT for 111 historical mixed cases of HNC. The second model (RP-RAD) was trained on plans optimized with RAD for the purpose of this study, using the same set of 111 historical cases.

An evaluation cohort of 52 consecutively treated, independent cases of OPC was identified and optimized with VMAT and RAD, respectively, using RP. Both the training- and the evaluation cohorts were previously treated at Karolinska University Hospital. The study was approved by the Swedish Ethical Review Authority (case ID: 2023-04515-01).

### 2.2 Planning study design

**Figure 1:**
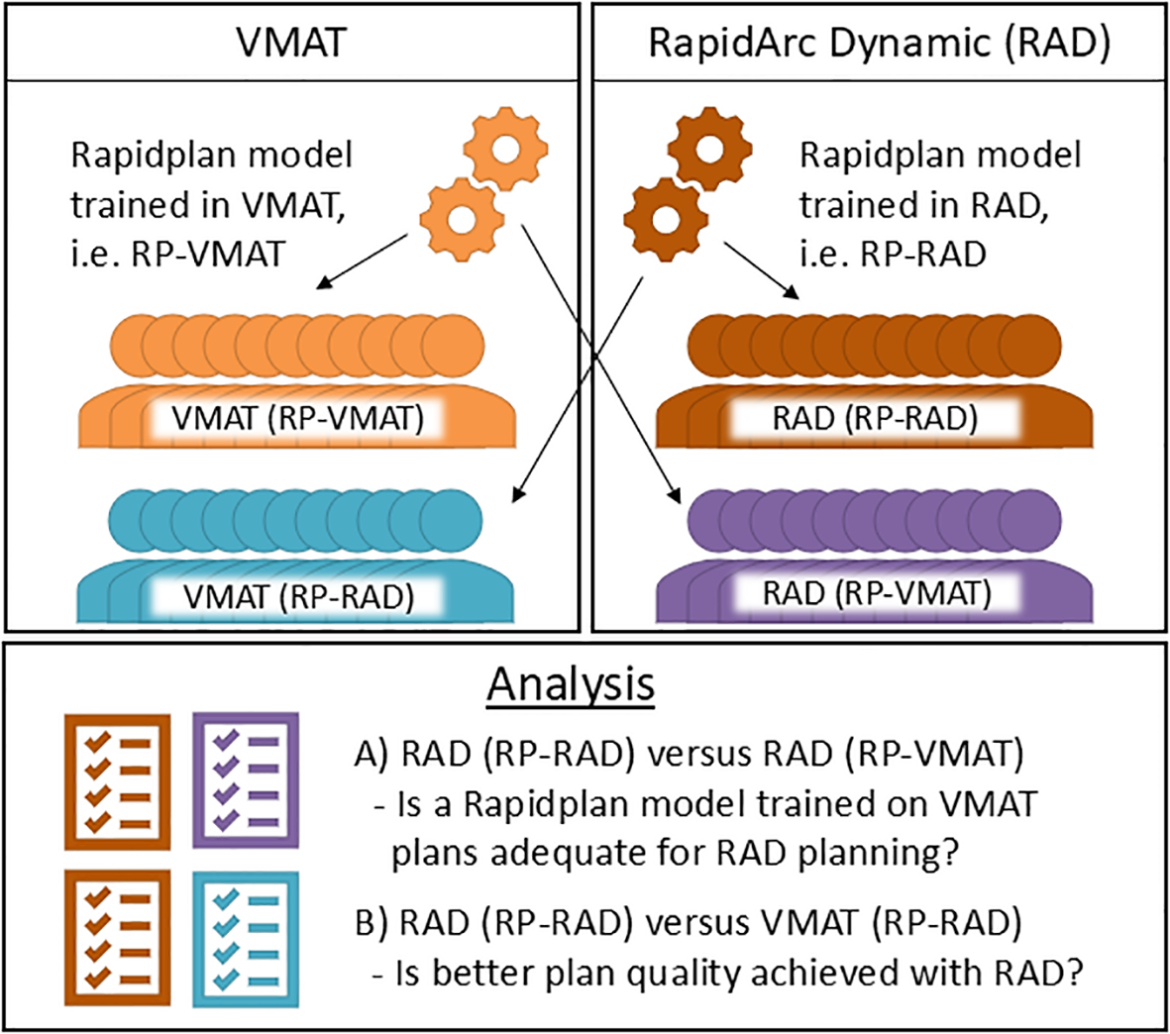
Planning study design: two RP models were used, trained on plans optimized with VMAT and RAD, respectively. In an independent evaluation cohort, each model was used to optimize both a VMAT plan and a RAD plan, resulting in four plans per case.

RATING (22) guidelines were considered when designing and evaluating this study (scoring sheet reported in the **Supplementary Materials**). **Figure 1** outlines the planning study design, including four plans for each evaluation case: 1) a VMAT plan optimized with RP-VMAT (VMAT (RP-VMAT)), 2) a VMAT plan optimized with RP-RAD (VMAT (RP-RAD)), 3) a RAD plan optimized with RP-VMAT (RAD (RP-VMAT)) and 4) a RAD plan optimized with RP-RAD (RAD (RP-RAD)). The comparison of RAD (RP-RAD) against RAD (RP-VMAT) aimed to evaluate the improvement in RAD plan quality when using a RP model re-trained for RAD. To verify that the quality of the generated RAD plans was at least equivalent to that of the current technique, VMAT (RP-VMAT) acted as a reference. More specifically, the comparison of RAD (RP-RAD) against VMAT (RP-RAD) aimed to evaluate the potential improvements in plan quality with the new degrees of freedom introduced with the RAD optimizer (the use of STAMPs and a dynamic collimator).

### 2.3 Planning techniques

**Figure 2:**
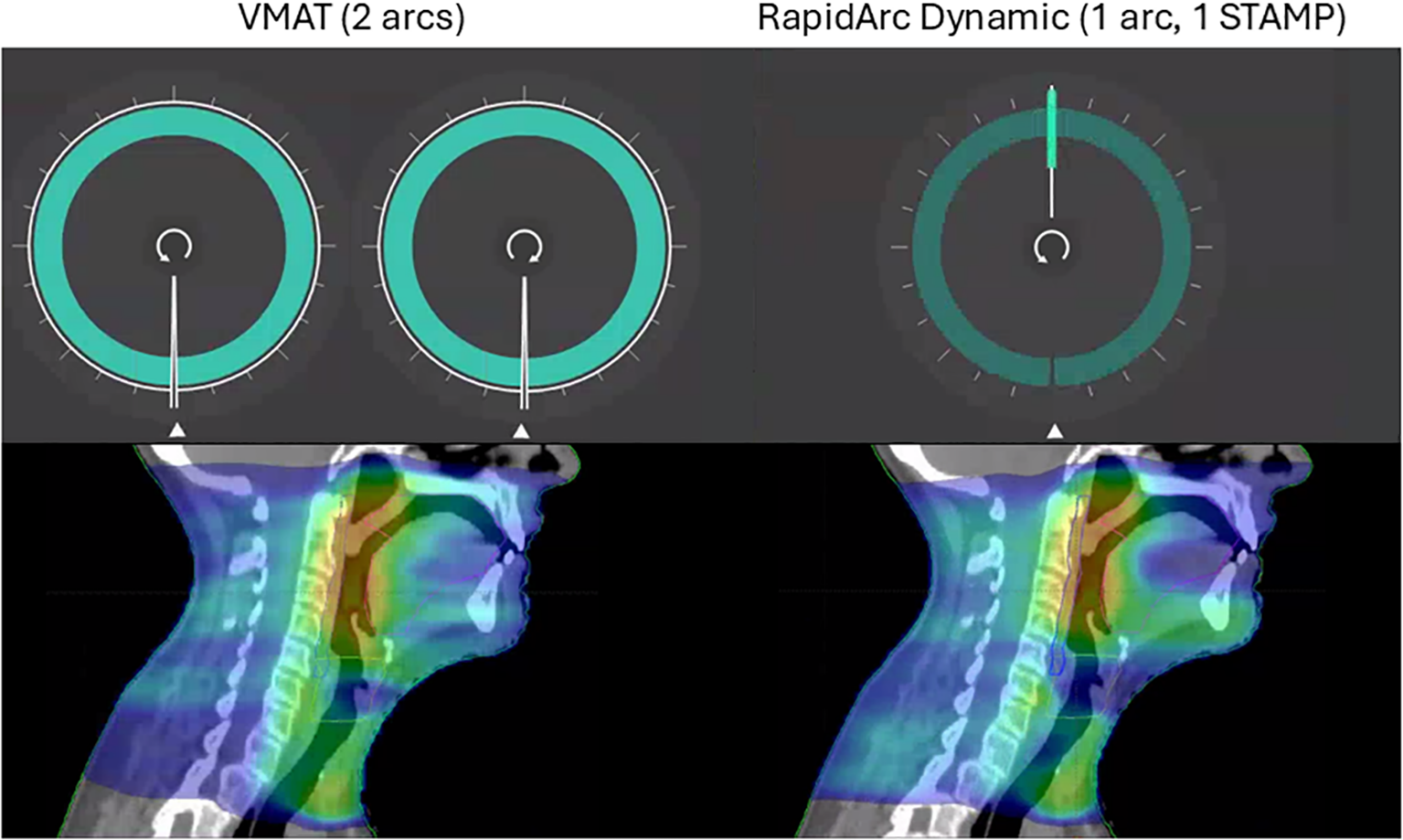
Beam arrangement for VMAT plans (left) and RAD plans (right), respectively, and example dose distributions for a case of tonsil cancer.

The planning technique was consistent throughout the study. The VMAT plans included two full arcs, usually at collimator angles of 15° and 345°, respectively (**Figure 2**-left). The RAD plans included a single full arc with one STAMP at gantry angle 0° and collimator angle 90°, with a dynamic collimator throughout the gantry rotation (**Figure 2**-right). The relative weight of the STAMP- and arc control-points, respectively, was set to the *balanced* option which assigned equal number of control points to the arc component and the STAMP component. The beam arrangement for the RAD plans was selected based on early experience with the technique, where an anterior STAMP with collimator angle of 90° was found to spare central OAR effectively, and a second arc was found to yield only a limited increase in plan quality. Reducing the number of arcs offers the potential to reduce the treatment delivery time, but may affect the plan complexity. Using the RAD optimizer, the chosen beam arrangement appeared to give similar or better plan quality compared to the established 2-arc VMAT technique, based on departmental clinical goals and a qualitative evaluation by an experienced HNC oncologist. In both cases, 6 MV beams were used and all plans were calculated using AcurosXB version 18.1 with the dose to medium option.

### 2.4 Planning aims

All plans were optimized by the same planner, for the purpose of the study, using clinical delineations of target structures and OAR and local clinical goals (**Supplementary Table 1**). A simultaneous integrated boost technique was used, with a prescription of 68 Gy (2 Gy per fraction) to the primary tumor and positive lymph nodes clinical target volume (CTV_68) (gross tumor volume (GTV) + 5 mm), 61.2 Gy (CTV_61.2) to an extended CTV volume around the primary tumor (GTV + 10 mm), and 51.68 Gy to the elective CTV (CTV_51.68). In some cases, CTV_61.2 was not applicable. Planning target volumes were defined by expanding each CTV by a 5 mm isotropic margin and cropped to the skin with a margin of 3 mm (to avoid the build-up region). All plans were required to fulfil the target coverage goals and the dose constraints for the spinal canal and brainstem.

For both VMAT and RAD, an initial optimization was performed using the applicable RP model (either RP-VMAT or RP-RAD), and where the mandatory goals were not achieved, manual tuning of the objectives was performed successively, over repeated re-optimization cycles, to comply with these. The RP models included line objectives and mean dose objectives, minimizing the dose to the parotid glands, submandibular glands, oral cavity, constrictor muscle, larynx, trachea and esophagus **(Supplementary Tables 2**, **3**). A ‘manual’ normal tissue objective was also included.

The RAD plans used for training RP-RAD were optimized by first applying RP-VMAT, and thereafter manually re-optimized repeatedly until further improvements in OAR and the normal-tissue objective could not be achieved, while fulfilling the mandatory goals for the target volumes, spinal canal and brainstem. RP models used in this study were validated using well reported methodologies (23).

### 2.5 Plan evaluation

The plans were evaluated with regards to plan quality, measured by the mean dose (*D_mean_*) to the parotid glands, submandibular glands, oral cavity, constrictor muscles, larynx, trachea and esophagus. The normal tissue complication probability (NTCP) was estimated using published models for severe late xerostomia (24), moderate late dysphagia (25) and severe acute oral mucositis (26), respectively. Model selection was inspired by the rating in Langendijk et al. (27). To calculate NTCP, dose-volume histograms (DVH) for the relevant OAR were extracted from the treatment planning system and converted to EQD2 using *α/β* = 3 Gy.

The VMAT- and RAD plans optimized with RP-VMAT were evaluated in terms of plan complexity and deliverability, exploring any changes compared to current clinical practice. Six complexity metrics were considered: the number of monitor units (MU), average aperture area (AAA), average aperture per leaf couple (ALPO), beam area (BA), beam modulation (BM) and beam irregularity (BI), as defined by Du et al. (28). Further, a subset of nine plans was measured with the Delta4 phantom (Scandidos, Uppsala, Sweden), and the gamma pass rates were compared for the two sets of plans (29). Initially, five cases were randomly selected for verification measurement and the correlation between the pass rate and all complexity metrics was evaluated for these. The complexity metric most strongly correlated with a low measurement pass rate was then used to identify the four most complex cases of the full cohort. Verification measurements were then performed also for these plans, completing the subset for delivery evaluation.

### 2.6 Statistical analysis

Descriptive statistics were reported. The difference in plan quality measures between sets of plans was determined using the related-samples Friedman’s two-way ANOVA test by ranks, with the significance level set to p = 0.05. A Bonferroni correction for multiple testing was applied, multiplying the p value by the number of tests, for plan quality metrics (*D_mean_* and NTCP) and complexity metrics, respectively. All analyses were performed in IBM SPSS Statistics 29.0 (IBM, NY, USA).

## 3 Results

Out of the 52 evaluation cases, four were excluded from the analysis: two presented technical problems, one had the wrong ICD code (actually treated for prostate cancer), and one had a large internal air volume, requiring non-standard planning. The characteristics of the 48 patients included in the planning study are listed in **Table 1**.

**Table 1:** Distribution of patient/plan characteristics of the final evaluation cohort.

Characteristics	Count
Tumor type	
Tonsil	23
Base of tongue	21
Oropharynx non-specified	2
Neck lymph node	1
Tonsil + larynx (2 targets)	1
Treatment of lymph nodes	
Bilateral	43
Unilateral	5
GTV-CTV expansion	
5 + 5 mm (2 dose levels)	47
10 mm (1 dose level)	1

All four plans, for all evaluation cases, fulfilled the target coverage goal of D98% ≥ 95% of the corresponding prescribed dose; VMAT plans optimized with RP-VMAT and RP-RAD needed manual tweaking in 17% and 73% of the cases to achieve coverage, and RAD plans optimized with RP-VMAT and RP-RAD were tweaked in 35% and 54% of the cases, respectively.

**Figure 3:**
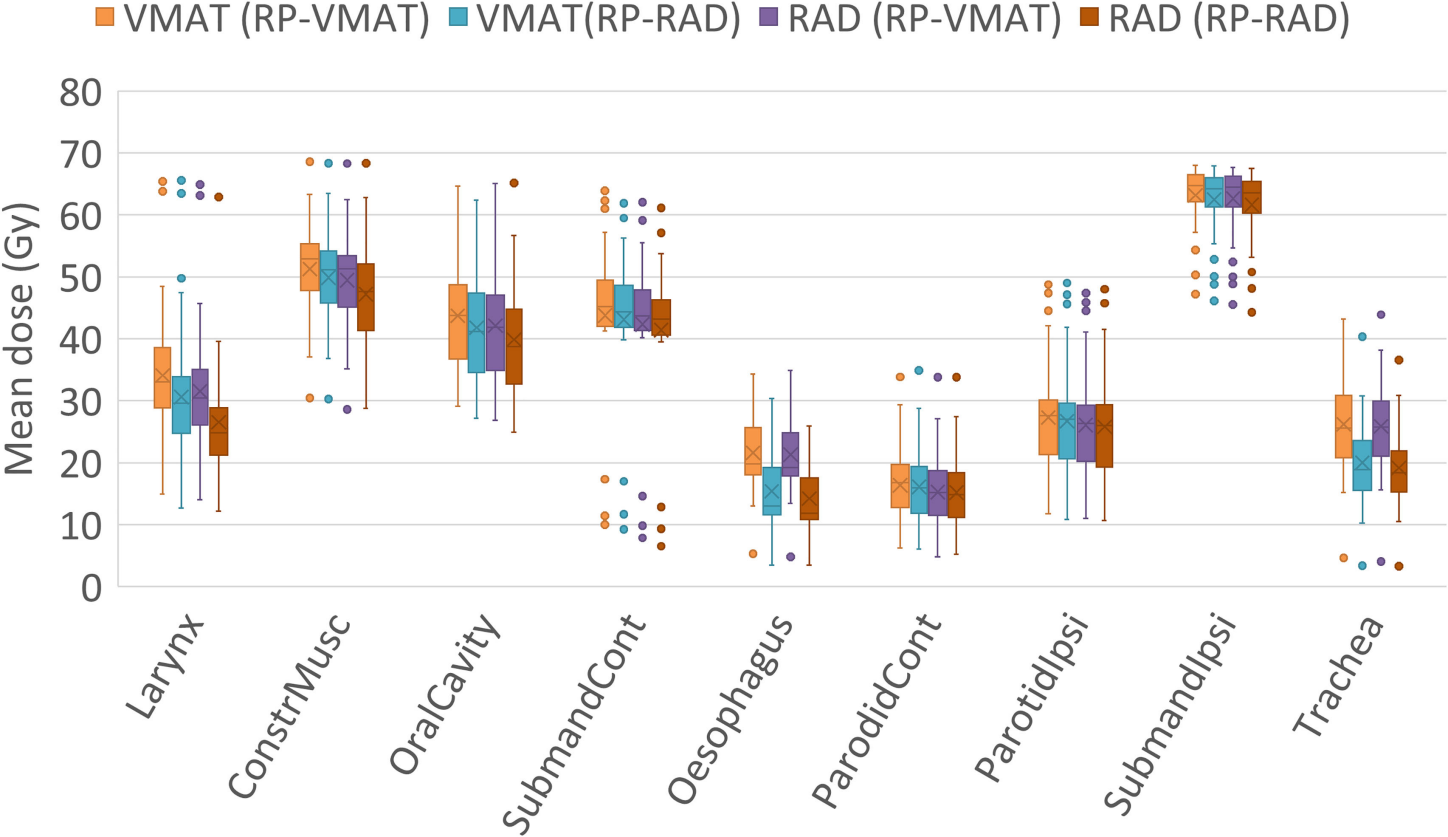
Mean dose to OAR for the evaluation cohort, ordered according to decreasing relative difference between the VMAT (RP-RAD) plans and RAD (RP-RAD) plans. The box indicates median values and the inter-quartile range, the ‘x’ indicates the mean value, and the whiskers 1.5 times the inter-quartile range. The dots are outliers.

For RAD plans, the RAD-based RP model improved the OAR sparing, as seen in **Supplementary Table 4**, **Figure 3**; the RAD (RP-RAD) plans significantly reduced *D_mean_* (median (IQR)) to all OAR compared to RAD (RP-VMAT), except the parotids, with the greatest reduction observed for the esophagus (7.1 (6.4-8.0) Gy) and trachea (6.7 (6.1-7.8) Gy). However, no OAR received a higher median *D_mean_* with RAD (RP-VMAT) compared to VMAT (RP-VMAT). Thus, the RAD plans were at least equivalent to the VMAT plans when optimizing with the current RP model (RP-VMAT), indicating the safety of initially implementing RAD with a VMAT-based RP model.

**Figure 4:**
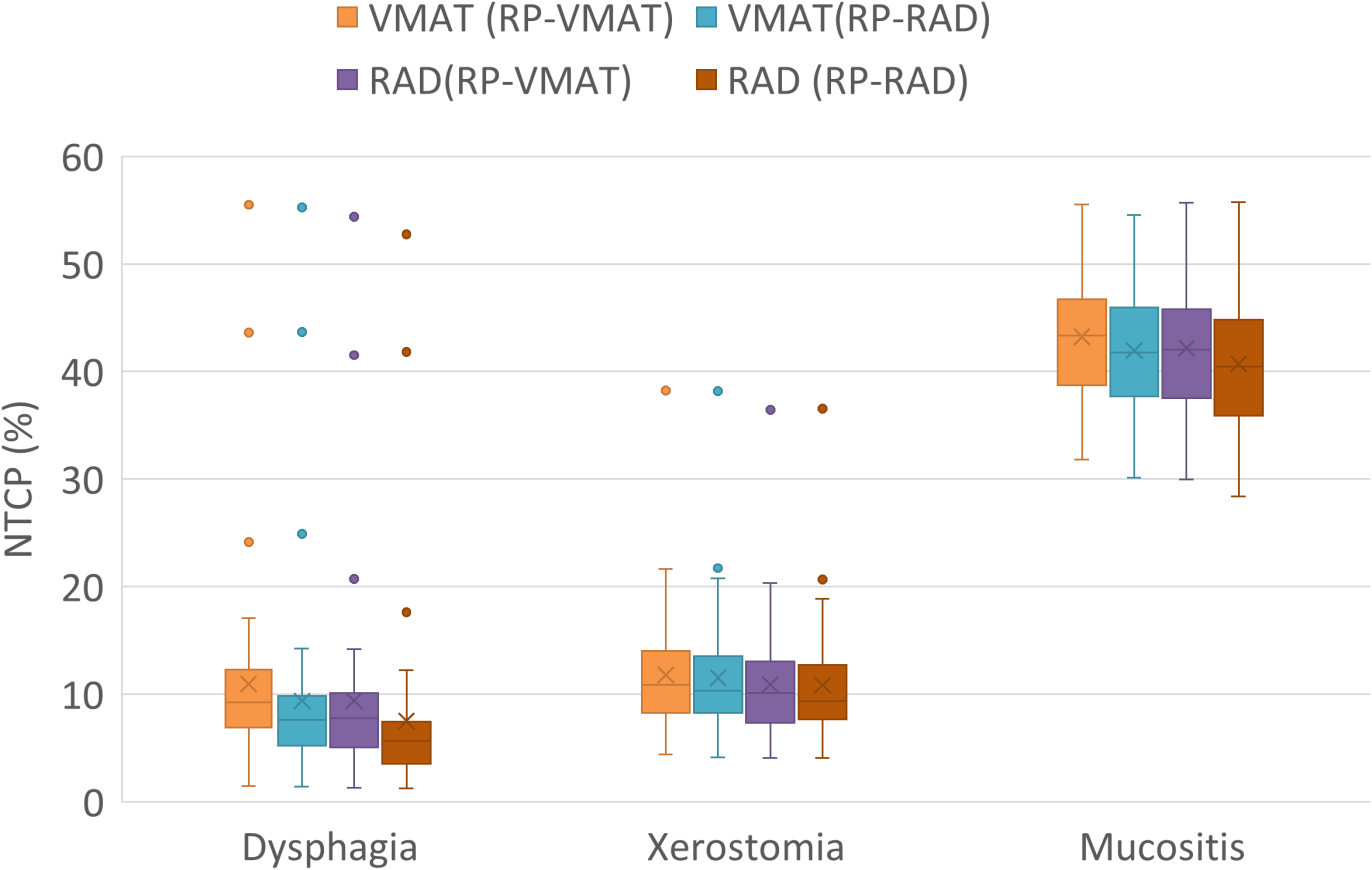
Estimated NTCP for the evaluation cohort: moderate late dysphagia (based on the *D_mean_* to larynx and constrictor muscle) (21), severe late xerostomia (based on the *D_mean_* to the total parotid volume) (20), and severe acute mucositis (based on the *D_mean_* to the oral cavity) (22). The box indicates median values and the inter-quartile range, the ‘x’ indicates the mean value, and the whiskers 1.5 times the inter-quartile range. The dots are outliers.

To evaluate the improvement in OAR sparing from the novel RAD solution, RAD- and VMAT plans optimized with the RAD-based RP model were compared, i.e. RAD (RP-RAD) vs. VMAT (RP-RAD). Here, all OAR except the trachea were significantly better spared with RAD (RP-RAD) (p = 0.039 for the esophagus, and p < 0.001 for all other OAR), with the greatest median reduction in *D_mean_* observed for the larynx and the constrictor muscle, amounting to 4.0 (2.3-5.2) Gy and 2.5 (1.6-3.4) Gy, respectively (**Figure 3**). The reduction in estimated NTCP was also significant for dysphagia and mucositis (p ≤ 0.001), when comparing RAD- and VMAT plans optimized with RP-RAD (**Supplementary Table 5**, **Figure 4**). In line with the significant reduction in mean dose to the larynx and constrictor muscle, the greatest reduction of 1.7 (1.1-2.3) pp (22%) was seen for the risk of moderate late dysphagia. The risk of severe acute mucositis was reduced by 0.6 (0.9-1-8) pp (2.7%).

**Figure 5:**
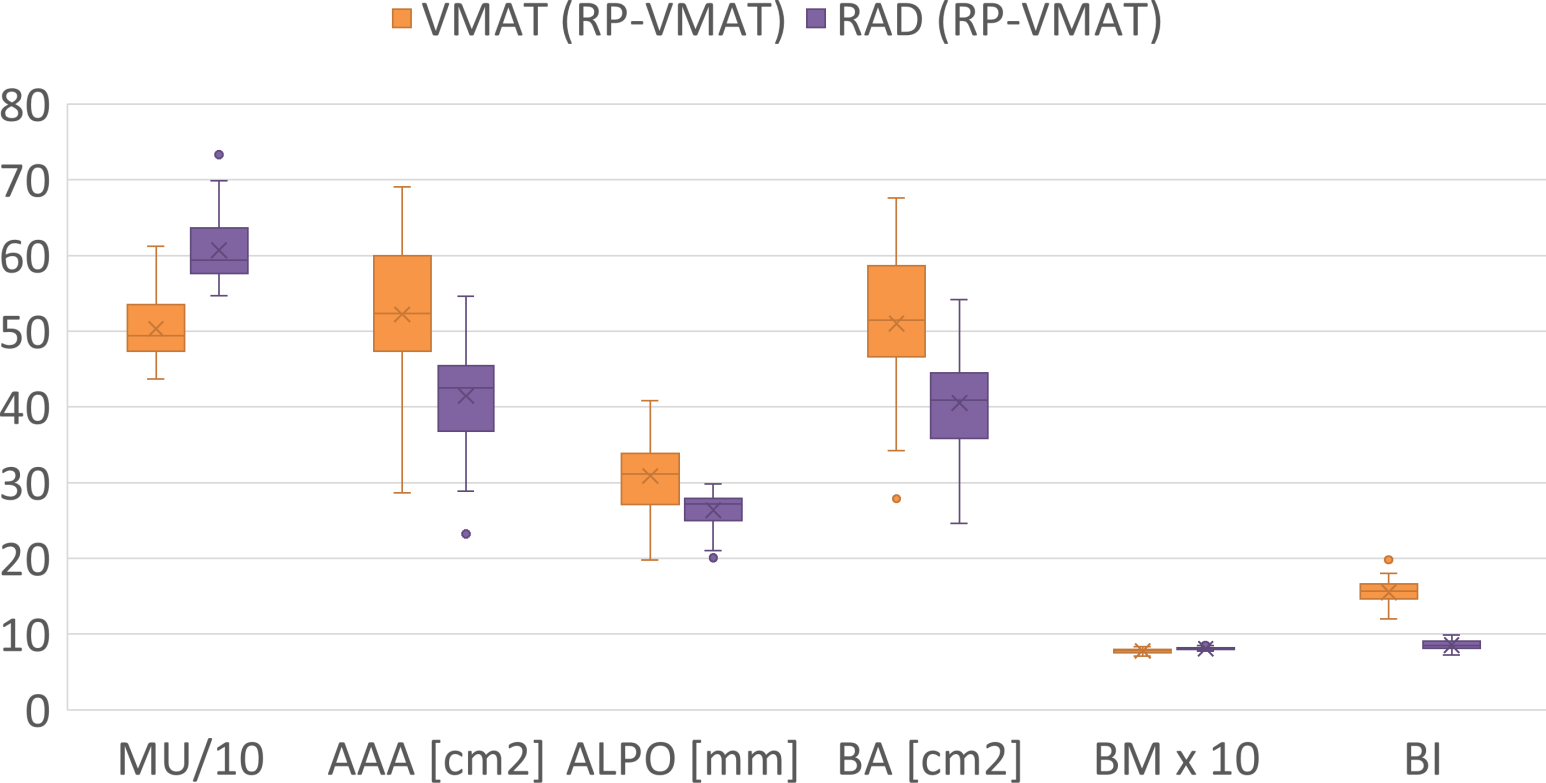
Complexity metrics for the whole evaluation cohort. Two metrics were transformed by a multiple of 10 for optimal visualization. MU. number of monitor units; AAA, average aperture area; ALPO, average leaf pair opening; BA, beam area; BM, beam modulation; BI, beam irregularity (28).

The VMAT (RP-VMAT) plans and the RAD (RP-VMAT) plans were compared in terms of complexity metrics (**Supplementary Tables 6**, **7**; **Figure 5**) for all cases, and in terms of deliverability for nine cases. The median number of MU increased by 100 for RAD plans compared to VMAT plans. Consistent with the higher number of MU, the RAD plans were significantly more complex than the VMAT plans, as measured by AAA, ALPO, BA and BM (p < 0.001). In contrast to these results, BI was lower for the RAD plans compared to the VMAT plans, indicating a lower degree of complexity.

The increased plan complexity in the RAD plans did not translate into a worse agreement between planned dose and the dose measured on the Delta4 phantom. For the nine verified cases, the median (IQR) gamma pass rates (2%/2 mm) were 98.5% (96.9-99.4%) and 98.3% (97.4-98.5%) for VMAT (RP-VMAT) and RAD (RP-VMAT) plans, respectively (p = 1.00).

While verifying the delivery of the selected plans, the delivery times were also recorded (**Supplementary Table 6**). The two arcs used for VMAT (RP-VMAT) resulted in a median (IQR) of 150 s (149–158 s), while the single arc with a static angle used for RAD (RP-VMAT) resulted in 81 s (78–84 s). The 46% reduction in delivery time between the two techniques was statically significant (p = 0.043).

## 4 Discussion

In this study, 48 sets of plans were evaluated using both the new RAD solution and the current VMAT solution, comparing the sparing of OAR achieved with a RAD-based- and a VMAT-based RP model. When optimized with RP-VMAT, the resulting RAD plans were equivalent to or superior to VMAT plans optimized with the same model. Due to its effectiveness (30), many clinics have come to rely heavily on RP for clinical treatment of HNC (9, 14, 31). Our findings suggest that centers adopting the RAD technique for OPC can begin planning with their existing VMAT-based RP models without compromising plan quality and OAR sparing, despite the added degrees of freedom (integration of STAMPs in an arc field and the dynamic collimator) and the new optimizer of RAD. However, open- or closed-loop validation is still recommended to ensure the suitability of each center’s RP model for their specific clinical cases.

Further, the current analysis showed improved OAR sparing with the new degrees of freedom offered by RAD. Hybrid strategies combining the modulation of IMRT and the arc delivery of VMAT have been previously explored (16, 32–35). In the work by Zhao et al. an hybrid solution has shown comparable target dose homogeneity and conformity to IMRT- or two-arc VMAT-only plans, while reducing dose to OAR (34). However, previous experience of the hybrid solution has come at the cost of efficiency due to the need of delivering the arcs and the IMRT component sequentially. In contrast, in the current study the newly released RAD solution showed a 46% reduction in treatment delivery time. This increase in efficiency and comparable plan quality have been possible in this early experience with RAD due to a streamlined beam arrangement of one full arc and a STAMP, integrated in one delivery sequence.

The improved OAR sparing with RAD in the current study promises an improvement in plan quality once RAD-based RP models replace VMAT-based RP models for RAD optimization. When both VMAT- and RAD plans were re-optimized with a RP-RAD model, *D_mean_* of midline structures was significantly reduced. This decrease in dose was reflected in the NTCP values which showed on average a significant reduction of the risk of dysphagia and mucositis. While the reduction did not reach the 5 pp level often considered when comparing photon- and proton plans (36), they were nonetheless considered of clinical interest, given the already low expected rate of moderate late dysphagia and the incremental development from VMAT to RAD. These results highlight the potential of RAD to impact clinical outcomes, through steep local dose gradients. However, with the RAD optimizer, the prescription isodose was less conformal in some places, where not pushed by OAR optimization objectives. This resulted in a streaking, ‘IMRT-like’, appearance of the isodose. This may require additional OARs to be considered in the optimization, and evaluation of high-dose volumes. Alternatively, the control points can be balanced more towards the arc component of the field, however potentially with less specific OAR sparing as a result.

The estimated NTCP values are indicative of the relative improvement with the novel RAD optimizer but should not be relied on numerically. As noted by Stieb et al. (37), the validation of these models should follow TRIPOD guidelines (38), and ideally the model would have to be validated externally. All of the NTCP models used in this work were validated either internally or externally and have been widely used in other studies (37, 39), however they were not validated on the cohort used in this study and a direct comparison was not performed in terms of demographics, disease profile and management. Additionally, there were differences in the way the larynx and the constrictor muscles were outlined compared to the development cohort for the moderate late dysphagia model (supraglottic larynx and superior pharyngeal constrictor muscle) (25).

By using RP models in the current planning study, user dependence was avoided in the comparison between VMAT- and RAD plans. The two RP models were developed using the same cohort, but while the RAD-RP model was trained specifically for the current study, the VMAT-RP model was developed for clinical use, with less previous experience of RP. Thus, the superiority of the RAD-RP model cannot be fully attributed to the potential of RAD planning, but also to better optimized training plans. This was partly achieved by the use of the VMAT-RP model when optimizing the RAD-RP training plans (40). On the other hand, a visual review of the RAD plans revealed some streaking of the prescription isodose into the mandible and the oral cavity, indicating a potential need for adapting RP models for RAD. The model is currently being updated to consider the high dose to small volumes of these OARs, as originally, primarily *D_mean_* was considered for the oral cavity and the mandible was not included as an OAR in the optimization. The impact on the other OAR by these modifications is indicated by preliminary results in the **Supplementary Materials**.

As the RAD solution includes both an arc- and a static gantry component, potentially increasing plan complexity, plan quality was also compared in terms of a selection of complexity metrics. As reported in the literature (39, 41), there is no consensus on which parameters should be used for this purpose, so a selection of typical metrics to assess VMAT and IMRT plans was used in this work. As RAD is a new technique, there is limited experience on which metric values to expect and the appropriateness of these metrics to assess the quality of the plans. Overall, in comparison to VMAT-only, the results for RAD showed a significant increase in complexity (in terms of AAA, ALPO, BA and BM). An average increase of 100 MUs was also found. This increase is in line with previously reported data that have shown a higher complexity for hybrid IMRT/VMAT plans in comparison to VMAT alone (33, 34). However, a selection of RAD plans was also measured on a linac to assess deliverability. No statistically significant differences between the techniques were found, despite using a stricter gamma criterion of 2%/2mm, similar to Antoine et al. (42). This demonstrates that the selected RAD plans, despite their increased complexity, could be accurately delivered for the cases studied.

## 5 Conclusions

In conclusion, existing VMAT-based RP models appear applicable when optimizing with the novel RAD solution, until new improved models can be developed. Further, the selected RAD beam arrangement, using a single arc and a single STAMP, shows promise for OPC in terms of efficiency and sparing of midline OARs.

## Data Availability

The raw data supporting the conclusions of this article will be made available by the authors, without undue reservation.
