# Supplementary material for: Can knowledge-based autoplanning keep up with advances in radiotherapy optimization for oropharyngeal cancer?
Source: Front Oncol. 2026 Mar 17;16:1755913. doi: 10.3389/fonc.2026.1755913 (PMC13035498; doi:10.3389/fonc.2026.1755913)
Supplement: Supplementary file 1 [file DataSheet1.pdf]

Supplementary Material

1 Clinical goals and planning objectives

Table 1. Clinical goals used in both the optimisation of the training plans, as well as the independent planning study plans

Structure	Clinical Goal
PTV_68	D98 > 64.6 Gy
	D2 < 71.4 Gy
PTV_61.2	D98 > 58.1 Gy
	D2 < 64.3 Gy
PTV_51.68	D98 > 49.1 Gy
	D2 < 54.3 Gy
Brain stem	$D_{0.1cc}$ < 60.0 Gy
Constrictor Muscle	D_{mean} < 50.0 Gy
Larynx	D_{mean} < 40.0 Gy
Mandible	D_{1cc} < 68.0 Gy
Oral Cavity	D_{mean} < 30.0 Gy
Parotid glands (individually, primary goal)	D_{mean} < 25.0 Gy
Parotid gland (contralateral, if ipsilateral exceeds primary goal)	D_{mean} < 20.0 Gy
PRV spinal canal	$D_{0.1cc}$ < 54.0 Gy
Spinal canal	$D_{0.1cc}$ < 50.0 Gy
Submandibular glands (individually)	D_{mean} < 39.0 Gy

Table 2. Optimisation objectives included in the RP-VMAT model

Structure	Optimisation Objective	Volume [%]	Dose	Priority
PTV_68	Upper	0	101%	130
	Lower	100	100%	155
PTV_61.2	Upper	0	101%	130
	Lower	100	100%	160
PTV_51.68	Upper	0	101%	130
	Lower	100	100%	150
Brain stem	Upper	2	54 Gy	90
	Upper (fixed vol., generated dose)	0	Generated	80
	Line (preferring OAR)	Generated	Generated	Generated
Constrictor muscle	Upper	0	100%	130
	Mean		Generated	60
	Line (preferring target)	Generated	Generated	60

Panettieri et al. Can knowledge-based autoplanning keep up with improved radiotherapy planning solutions in oropharyngeal cancer? Front. Oncol. 16:1755913

Oesophagus	Upper	0	100%	130
	Mean		Generated	60
	Line (preferring target)	Generated	Generated	60
Larynx	Upper	0	100%	130
	Mean		Generated	60
	Line (preferring target)	Generated	Generated	65
Oral cavity	Upper	0	100%	130
	Mean		Generated	60
	Line (preferring target)	Generated	Generated	60
Parotid glands	Upper	0	100%	130
	Mean		Generated	60
	Line (preferring target)	Generated	Generated	70
PRV spinal canal	Upper	0	45 Gy	130
	Upper (fixed vol., generated dose)	0	Generated	80
	Line (preferring OAR)	Generated	Generated	60
Spinal canal	Upper	0	43 Gy	130
	Upper (fixed vol., generated dose)	0	Generated	80
	Line (preferring OAR)	Generated	Generated	60
Submandibular glands	Upper	0	100%	130
	Mean		Generated	60
	Line (preferring target)	Generated	Generated	40
Trachea	Upper	0	100%	130
	Mean		Generated	60
	Line (preferring target)	Generated	Generated	60

Table 3. Optimisation objectives included in the RP-RAD model

Structure	Optimisation Objective	Volume [%]	Dose	Priority
PTV_68	Upper	0	101%	135
	Lower	100	100%	165
PTV_61.2	Upper	0	101%	135
	Lower	100	100%	170
PTV_51.68	Upper	0	101%	135
	Lower	100	100%	160
Brain stem	Upper	2	54 Gy	90
	Upper (fixed vol., generated dose)	0	Generated	80
	Line (preferring OAR)	Generated	Generated	Generated
Constrictor muscle	Upper	0	100%	130
	Mean		Generated	65
	Line (preferring target)	Generated	Generated	65

Oesophagus	Upper	0	100%	130
	Mean		Generated	65
	Line (preferring target)	Generated	Generated	65
Larynx	Upper	0	100%	130
	Mean		Generated	65
	Line (preferring target)	Generated	Generated	70
Oral cavity	Upper	0	100%	130
	Mean		Generated	75
	Line (preferring target)	Generated	Generated	65
Parotid glands	Upper	0	100%	130
	Mean		Generated	65
	Line (preferring target)	Generated	Generated	75
PRV spinal canal	Upper	0	45 Gy	130
	Upper (fixed vol., generated dose)	0	Generated	80
	Line (preferring OAR)	Generated	Generated	50
Spinal canal	Upper	0	43 Gy	130
	Upper (fixed vol., generated dose)	0	Generated	80
	Line (preferring OAR)	Generated	Generated	50
Submandibular glands	Upper	0	100%	130
	Mean		Generated	65
	Line (preferring target)	Generated	Generated	60
Trachea	Upper	0	100%	130
	Mean		Generated	65
	Line (preferring target)	Generated	Generated	65

2 Detailed results

Table 4. Mean dose (Gy) for the evaluation cohort plans: median (inter-quartile range, IQR), [minumum, maximum]. P values from related-samples Friedman's Two-Way ANOVA by Ranks tests, with Bonferroni correction for multiple tests.

	Median (IQR)				p			
	1: VMAT (RP-VMAT)	2: VMAT (RP-RAD)	3: RAD (RP-VMAT)	4: RAD (RP-RAD)	4 vs. 1	4 vs. 2	4 vs. 3	3 vs. 1
Larynx	33 (29-38) [14.9,65.4]	30 (25-34) [12.7,65.6]	30 (27-35) [14.0,64.9]	25 (21-29) [12.1,63.4]	**<0.001**	**<0.001**	**<0.001**	**<0.001**
Oesophagus	20 (18-25) [5.3,34.3]	13 (12-18) [3.5,30.4]	19 (18-24) [4.8,34.9]	12 (11-17) [3.5,25.9]	**<0.001**	**0.039**	**<0.001**	1.00
Constrictor muscle	53 (48-55) [30.4,68.6]	51 (46-54) [30.3,68.4]	51 (45-53) [28.6,68.3]	48 (42-52) [28.8,68.3]	**<0.001**	**<0.001**	**0.001**	**<0.001**
Oral cavity	44 (37-49) [29.1,64.7]	41 (35-47) [27.2,62.4]	42 (35-47) [26.8,65.0]	39 (33-45) [24.9,65.2]	**<0.001**	**<0.001**	**<0.001**	**<0.001**
Parotid Ipsilateral	28 (21-30) [11.7,48.8]	27 (21-29) [10.8,49.0]	26 (20-29) [11.0,47.4]	26 (20-29) [10.7,48.0]	**<0.001**	**<0.001**	1.00	**<0.001**
Parotid Contralateral	17 (13-20) [6.3,33.9]	16 (12-19) [6.0,34.9]	15 (12-19) [4.8,33.8]	15 (11-18) [5.2,33.8]	**<0.001**	**<0.001**	1.00	**<0.001**
Submandibular Ipsi	65 (62-66) [47.2,68.0]	64 (62-66) [46.1,67.9]	64 (61-66) [45.6,67.7]	64 (61-65) [44.3,67.5]	**<0.001**	**<0.001**	**<0.001**	**0.003**
Submandibular Cont	45 (42-49) [10.0,63.9]	44 (42-49) [9.2,62.5]	44 (41-48) [7.9,62.8]	43 (41-46) [6.5,61.7]	**<0.001**	**<0.001**	**0.001**	**<0.001**
Trachea	26 (21-31) [4.6,43.2]	19 (16-23) [3.4,40.3]	26 (22-29) [4.1,43.9]	18 (15-22) [3.3,36.5]	**<0.001**	0.859	**<0.001**	1.00

Table 5. NTCP (%) for the evaluation cohort plans: median (inter-quartile range, IQR). P values from related-samples Friedman's Two-Way ANOVA by Ranks tests, with Bonferroni correction for multiple tests.

	Median (IQR)				p			
	1: VMAT (RP-VMAT)	2: VMAT (RP-RAD)	3: RAD (RP-VMAT)	4: RAD (RP-RAD)	4 vs. 1	4 vs. 2	4 vs. 3	3 vs. 1
Xerostomia	11 (8.4-14) [4.4,38.2]	10 (8.4-14) [4.1,38.2]	10 (7.6-13) [4.1,36.4]	9.3 (7.7-13) [4.1,36.5]	**<0.001**	1.00	**<0.001**	**<0.001**
Dysphagia	9.2 (6.9-12) [1.5,55.5]	7.6 (5.3-9.7) [1.4,55.3]	7.8 (5.3-10) [1.3,54.4]	5.7 (3.5-7.3) [1.3,52.7]	**<0.001**	**<0.001**	**<0.001**	**<0.001**
Mucositis	43 (39-47) [31.8,55.5]	42 (38-46) [30.1,54.5]	42 (38-46) [30.0,55.7]	41 (36-45) [28.4,55.8]	**<0.001**	**<0.001**	**<0.001**	**<0.001**

Table 6. Complexity metrics, verification measurement pass rate (2% / 2 mm) and delivery times: median (inter-quartile range, IQR). P values from related-samples Friedman's Two-Way ANOVA by Ranks tests, with Bonferroni correction for multiple tests. Complexity metrics correspond to the full cohort, while the verification pass rates and delivery times correspond to a subset of 9 cases.

	VMAT (RP-VMAT)	RAD (RP-VMAT)	p
MU	494 (475-535) [437,612]	594 (578-635) [547,733]	**<0.001**
AAA (cm^2)	52 (48-59) [29,69]	43 (38-45) [23,55]	**<0.001**
ALPO (mm)	31 (27-34) [20,41]	27 (25-28) [20,30]	**<0.001**
BA (cm^2)	51 (47-59) [28,68]	41 (36-44) [25,54]	**<0.001**
BM	0.78 (0.76-0.80) [0.71,0.83]	0.81 (0.80-0.82) [0.77,0.86]	**<0.001**
BI	16 (15-17) [12,20]	8.5 (8.1-9.0) [7.2,9.9]	**<0.001**
Pass rate (2%/2mm) (%)	98.5 (96.9-99.4) [95.2,99.4]	98.3 (97.4-98.5) [96.4,99.5]	1.00
Delivery time (s)	150 (149-158) [144,190]	81 (78-84) [77,97]	**0.043**

Table 7. Complexity metrics, verification measurement pass rate and delivery times for the subset of 9 cases. R1-R5 were randomly selected for measurement, and C1-C4 were selected as those with the highest number of monitor units.

	R1	R2	R3	R4	R5	C1	C2	C3	C4
MU									
VMAT (RP-VMAT)	480	475	453	549	508	509	476	543	471
RAD (RP-VMAT)	574	576	577	579	593	650	651	688	726
AAA (cm^2)									
VMAT (RP-VMAT)	42	38	60	41	54	62	68	57	51
RAD (RP-VMAT)	36	30	45	36	45	45	44	46	39
ALPO (mm)									
VMAT (RP-VMAT)	26	23	32	26	30	35	39	31	32
RAD (RP-VMAT)	25	23	27	25	28	27	27	28	26
BA (cm^2)									
VMAT (RP-VMAT)	42	37	60	40	52	60	67	55	51
RAD (RP-VMAT)	35	31	45	36	44	44	45	43	31
BM									
VMAT (RP-VMAT)	0.80	0.76	0.76	0.80	0.79	0.76	0.71	0.79	0.77
RAD (RP-VMAT)	0.82	0.83	0.82	0.81	0.80	0.81	0.79	0.80	0.86
BI									
VMAT (RP-VMAT)	15	15	17	18	16	13	12	17	15
RAD (RP-VMAT)	8.1	8.7	9.2	8.2	8.3	9.7	9.0	9.0	8.0
Pass rate (2%/2mm) (%)									
VMAT (RP-VMAT)	99.4	96.9	99.4	98.5	99.0	95.2	98.1	99.4	96.8
RAD (RP-VMAT)	98.5	98.1	99.3	97.4	98.3	96.4	98.5	99.5	97.4
Delivery time (s)									
VMAT (RP-VMAT)	150	150	190	160	158	156	149	144	149
RAD (RP-VMAT)	81	78	79	78	81	97	84	77	85

3 Adaptation of the RP-RAD model for clinical use

In the RP models in the current study, no objective was included for the mandible, since the experience from VMAT was that the natural conformality of the high isodoses typically spared this OAR sufficiently. Thus, the mandible was not systematically delineated, neither for the training cases, nor for the independent evaluation cases. However, when evaluating the RAD plans, it was discovered that the high isodoses were sometimes less conformal compared to VMAT, and there was frequently a streaking, 'IMRT-like', appearance. This raised a concern that the RP-RAD model might not spare the mandible sufficiently. In addition, in cases with the target located adjacent to the oral cavity, a larger volume of high dose to this OAR might be a concern if only D_{mean} is considered in the optimisation. To address these concerns, the RP-RAD model evaluated in the current analysis, is now being updated before taking it into clinical use. The mandible was outlined where missing, and all training cases were optimised using RP-RAD, and manually tweaked based on the following objectives:

- Mandible D_{1cc}, D_{5cc} and $D_{30\%}$
- Oral cavity V_{50Gy}

In addition, target coverage was given a slightly higher priority, compared to RP-RAD, to avoid a frequent need for manual tweaking (see Results in the main manuscript).

Ten test cases, with the largest overlap of the mandible by the 95% isodose in the RAD (RP-RAD) plans, were selected to evaluate the updated model, hereafter referred to as RP-RADclin. Table 7 and Figure 1 show the OAR doses for the test cases when optimised with RAD (RP-RAD) and RAD (RP-RADclin), respectively. These results show that the mandible D1cc was significantly reduced with the RP-RADclin model, while there was no significant change to any other OAR.

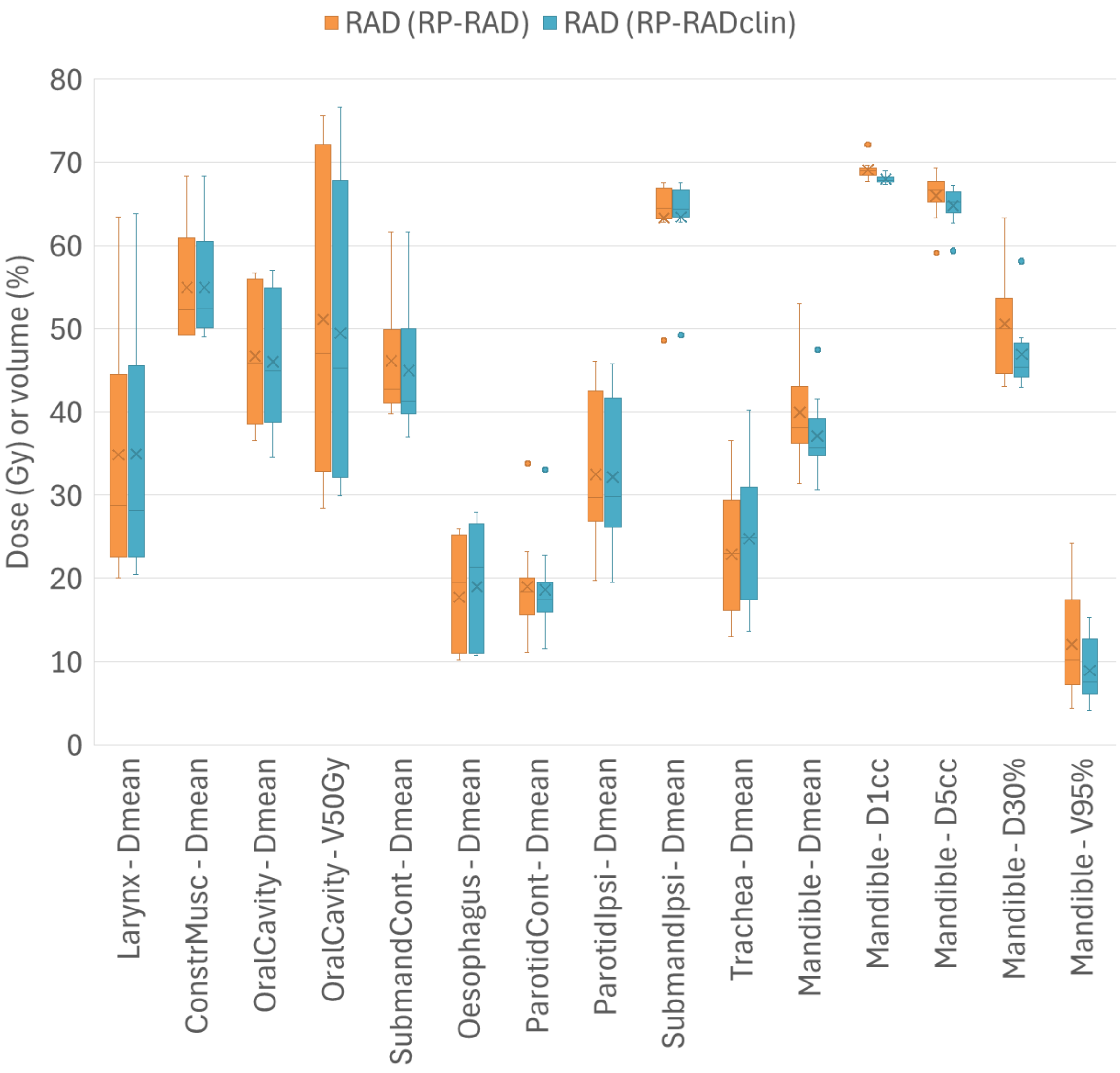

Figure 1. OAR doses in RAD (RP-RAD) plans and RAD(RP-RADclin) plans, respectively, for 10 test cases. Sorting consistent with Figure 2 in the main manuscript.

Table 8. OAR doses in RAD (RP-RAD) plans and RAD(RP-RADclin) plans, respectively, for 10 test cases; mean (standard deviation). P values from Wilcoxon signed-rank tests.

	RAD (RP-RAD)	RAD (RP-RADclin)	p
Larynx			
D_{mean} (Gy)	29 (23-38)	28 (23-39)	0.91
Constrictor muscle			
D_{mean} (Gy)	52 (50-59)	52 (50-58)	0.91
Oral Cavity			
D_{mean} (Gy)	46 (40-55)	45 (39-54)	0.91
V_{50Gy} (%)	47 (34-69)	45 (33-65)	0.76
Oesophagus			
D_{mean} (Gy)	20 (12-25)	21 (11-27)	0.49
Parotid Ipsilateral			
D_{mean} (Gy)	30 (28-39)	30 (27-38)	0.91
Parotid Contralateral			
D_{mean} (Gy)	18 (16-19)	18 (17-18)	0.65
Submandibular Ipsi			
D_{mean} (Gy)	64 (64-67)	64 (64-66)	0.92
Submandibular Cont			
D_{mean} (Gy)	43 (41-47)	41 (40-47)	0.43
Trachea			
D_{mean} (Gy)	23 (18-29)	25 (21-31)	0.55
Mandible			
D_{mean} (Gy)	38 (36-41)	36 (35-38)	0.14
D_{1cc} (Gy)	69 (69-69)	68 (68-68)	**<0.01**
D_{5cc} (Gy)	67 (66-68)	65 (64-66)	0.12
$D_{30\%}$ (Gy)	50 (46-52)	45 (44-48)	0.14
$V_{95\%}$ (%)	10 (8.4-16)	7.6 (6.7-12)	0.27

RATING score sheet

#	Question	Points	Applicable/ relevant	Answer yes
	**Questions for the Introduction**			
	The study aim formulated by research questions			
1	Does the study have a concise and precise study aim, defined with a restricted number of interconnected questions?	10		☑
	The motivation for the research questions			
2	Has relevant up to date literature been included to support the need for the current study?	5		☑
3	Does the study address an existing knowledge gap?	10		☑
	**Questions for Materials and Methods**			
4	Is the global study design adequate for answering the posed research questions?	10		☑
5	Is the global study design described in sufficient detail for others to interpret and reproduce the results?	5		☑
	Patient cohort			
6	Are the inclusion and exclusion criteria of the patient cohort described?	1	☑	☑
7	Is the clinical patient information of the cohort presented, including disease type, site(s) and clinical staging?	1	☑	☑
8	Is the included number of patients stated, explained and justified?	1	☑	☑
9	Has there been consideration of the need for ethical and/or legal approval for the study and if needed, is there a statement about this?	5		☑
	Imaging procedures			
10	Have the scanning parameters been reported in sufficient detail (image modalities, equipment model, slice thickness, voxel size, patient position (e.g. head first, supine, etc.) etc.)?	1	☐	☐
11	Has the applied immobilisation equipment been described, (e.g. vendor and type, standard settings, etc.) where relevant?	1	☐	☐
	Treatment machine and settings			
12	Have the treatment machine and relevant parameters been described with sufficient detail (model, beam energy, MLC, etc.)?	1	☑	☑
13	Have the monitor unit reference conditions been defined, where relevant?	1	☐	☐
	Definition of targets and OARs			
14	Has GTV definition been described in sufficient detail, with references if possible?	1	☑	☑
15	Has CTV definition been described in sufficient detail, with references if possible?	1	☑	☑

16	Has the establishment of PTVs (or alternatively robustness settings) been described in sufficient detail?	1	☑	☑
17	Have PTV sizes in the patient cohort been described?	1	☐	☐
18	Have OAR definitions been described in sufficient detail, with references if possible?	1	☑	☑
19	Have PRV margins been described in sufficient detail, with references if available?	1	☑	☐
	Treatment planning system and dose calculation			
20	Have all applied dose calculation algorithms been described in sufficient detail?	1	☑	☑
21	For any commercial software used, have the manufacturer, algorithms and specific versions been stated?	1	☑	☑
22	Have all relevant user parameters and settings in the TPS been reported, e.g. beams, dose grid, control point spacing?	1	☑	☑
23	Have all volumes been evaluated with the same software/methodology?	1	☑	☑
	Planning aims and optimisation			
24	Are clear planning aims defined, including imposed hard constraints and planning objectives (with or without soft constraints)?	5		☑
25	Has the ranking of planning objectives (priorities) been described?	5		☐
26	Is the dose prescription clearly defined?	10		☑
27	Is there a narrative description of the applied optimisation process, including the handling of all objectives with their ranking?	5		☑
28	If manual intervention during or after optimisation is allowed, has this been described?	1	☑	☑
	Bias mitigation			
29	Have enough study details been provided such that bias issues could be noted?	5		☑
30	Has bias been sufficiently mitigated to reliably answer the posed research question?	10		☑
	Plan acceptability – minor and major protocol deviations			
31	Was the procedure for assessment of plan acceptability well-described?	1	☑	☑
32	Was the procedure for assessment of minor and major protocol deviations well described?	1	☐	☐
	Plan (re-)normalisation for plan comparisons		☑	☑
33	Has plan (re-)normalisation been described sufficiently?	1		
	Dose-volume parameters for plan evaluation and comparison			
34	Have sufficiently comprehensive dose-volume parameters been used for plan evaluations and comparisons?	5		☑
	Population-mean DVHs			

35	Has the algorithm for creating population-mean/median DVHs been reported?	1	☐	☐
36	Have the definitions of confidence intervals been included?	1	☐	☐
	Plan evaluations by clinicians			
37	Have clinicians scored plans to assess quality?	1	☐	☐
38	Were plan comparisons by clinicians blinded?	1	☐	☐
	Predicted tumour control probability and normal tissue complication probabilities for plan evaluation and comparison			
39	Have any applied TCP models been described and referenced?	1	☐	☐
40	Have any applied NTCP models been described and referenced?	1	☑	☑
	Plan deliverability and complexity			
41	Have methods used to assess plan deliverability and complexity been described in sufficient detail?	1	☑	☑
	Composite plan quality metrics			
42	Is there a sufficient basis (e.g. in the literature) for any selected composite plan quality metrics?	1	☐	☐
43	Is there an adequate description of the calculation of the composite plan quality metrics?	1	☐	☐
	Planning and delivery times			
44	Has measurement of planning times been described in sufficient detail?	1	☐	☐
45	Has the establishment of delivery times been described in sufficient detail?	1	☑	☑
	Statistical analysis			
46	Have proper statistical methods been used and described in sufficient detail?	5		☑
47	In case of multiple testing for research questions, has this been handled appropriately?	1	☑	☑
	**Questions for Results**			
48	Does the provided data contribute to (at least partly) answering all aspects of the research questions, e.g. plan acceptability, dosimetric quality, deliverability and planning and delivery times?	10		☑
	Dose distribution reporting			
49	Are complete summaries of the dose distributions in the patient cohort provided (low doses, high doses, OARs, PTV, patient, etc.)?	5		☑
50	Are tables and figures optimised to clearly present the results obtained?	1	☑	☑
51	Have the answers to the research questions been illustrated for an example patient by providing dose distributions, DVHs, etc.?	1	☑	☑
	Plan acceptability reporting – minor and major protocol deviations			

52	In case of treatment technique or planning technique comparisons, was plan acceptability reported separately for each technique?	1	☑	☑
53	Has plan acceptability been reported in sufficient detail: how many plans were acceptable, how many were not and for what reasons (e.g. violation of hard constraints, violation of soft constraints, other reasons)?	1	☐	☐
54	Was there adequate reporting of minor and major protocol deviations?	1	☐	☐
	Deliverability and complexity reporting		☑	☑
55	Has the deliverability of the plans been adequately reported?	1	☑	☑
56	Have plan deliverability and complexity been investigated in sufficient detail in relation to the posed research questions?	1		
	Planning and delivery times reporting			
57	Have planning and delivery times been adequately evaluated and reported?	1	☐	☐
	Patient-specific analyses reporting			
58	Is there sufficient description of inter-patient variations in the results presented?	1	☑	☑
59	Have outlier patients been reported and has any exclusion from population analyses been sufficiently motivated and explained?	1	☐	☐
	Statistical reporting		☑	
60	Are the p-values reported appropriately?	1	☑	☑
61	Are there confidence intervals for the appropriate parameters?	1		☑
**Questions for discussions**				
62	Is there an overall interpretation of the data presented in the Results section as to how the posed research questions are answered?	10		☑
	Comparison with literature			
63	Has the study been sufficiently discussed in the context of existing literature?	5		☑
	Clinical and statistical significance		☑	☑
64	Does the discussion focus on statistically significant results?	1		☑
65	Is the potential clinical significance of the results clearly discussed (assuming practical application would be feasible)?	5		
	Clinical applicability of the study		☑	☑
66	Is future the clinical applicability sufficiently discussed?	1		
	Study limitations			

67	Has the impact of the study limitations on the provided answers to the research questions been sufficiently discussed?	10		☑
	Future work		☑	☑
68	Has the potential future work arising from the study been discussed?	1		
**Questions for conclusions**				
69	Do the presented conclusions represent answers to the posed research questions?	5		☑ ☑
70	Are the conclusions fully supported by the results?	5		☑
71	Are the conclusions a fair summary of all results?	5		
**Questions for supplementary**				
	Supplementary materials			
72	Is the information presented in the supplementary material of sufficient relevance?	1	☑	☑
73	Is the presentation of the included information of sufficient quality, including readability?	1	☑	☑
74	Has sufficient underlying data been made available or a willingness to share data been indicated, within local data sharing restrictions?	5		☑
**RATING remarks**				☑
75	Is the RATING score added to the manuscript?	5		☑
76	Is the accompanying question table added to the cover letter or the supplementary material?	1	☑	

RATING score	97%		
**RATING fraction**	193	of	199
